# Acknowledgement to Reviewers of the *Journal of Cardiovascular Development and Disease* in 2014

**DOI:** 10.3390/jcdd2010001

**Published:** 2015-01-09

**Authors:** JCDD Editorial Office

**Affiliations:** MDPI AG, Klybeckstrasse 64, CH-4057 Basel, Switzerland

The editors of the *Journal of Cardiovascular Development and Disease* would like to express their sincere gratitude to the following reviewers for assessing manuscripts in 2014:

Accornero, Federica
António Belo, José
Asirvatham, Samuel J
Bakkers, Jeroen
Belski, Regina
Blankesteijn, W. Matthijs
Blum, Martin
Boletta, Alessandra
Brewer, Alison C.
Campione, Marina
Chisaka, Osamu
Chiu, Sally
Cole, Mark
Cripps, Richard
Czubryt, Michael P.
Fedorov, Vadim V
Finberg, Robert W.
Foley, Julie F.
Goodship, Judith
Groot, A.C. Gittenberger-de
Hinton, Robert
Ichihara, S.
Jagla, Krzystof
Jiao, Kai
Kelly, Robert
Kimura, Wataru
Kühl, Michael
Lam, Jason T.
Latif, Najma
Lazzeri, Chiara
Lincoln, Joy
Mader, Robert
Männer, Jörg
Nikolaev, Viacheslav
Poelmann, Robert
Porrello, Enzo R.
Scambler, Peter
Schenke-Layland, Katja
Sedemera, David
Sollerbrant, Kerstin
Sugi, Yukiko
Torres Sánchez, Miguel
Volk, Talila
Wachten, Dagmar
Weiss, Robert M.
Xin, Mei
Yutzey, Katherine
Zabner, Joseph

